# Characterization of the complete chloroplast genome of *Pterygocalyx volubilis* (Gentianaceae)

**DOI:** 10.1080/23802359.2019.1640644

**Published:** 2019-07-16

**Authors:** Jiuli Wang, Qian Cao, Kezhou Wang, Rui Xing, Lirong Wang, Dangwei Zhou

**Affiliations:** a Key Laboratory of Biotechnology and Analysis and Test in Qinghai-Tibet Plateau, College of Ecological Environment and Resources, Qinghai Nationalities University, Xining, China;; b Key Laboratory of Adaptation and Evolution of Plateau Biota, Northwest Institute of Plateau Biology, Chinese Academy of Sciences, Xining, China

**Keywords:** *Pterygocalyx volubilis*, chloroplast genome, subtribe Swertiinae, Gentianaceae

## Abstract

*Pterygocalyx volubilis* Maxim. (Gentianaceae) is a traditional Chinese medicine, and its whole grass is used in the treatment of pulmonary tuberculosis and other conditions. Here, the complete chloroplast genome sequence of *P. volubilis* was reported based on the Illumina HiSeq Platform. The chloroplast genome genome is 154,365 bp in length, containing a pair of inverted repeated (IR) regions (25,928 bp) that are separated by a large single copy (LSC) region of 84,033 bp, and a small single copy (SSC) region of 18,476 bp. Moreover, a total of 130 functional genes were annotated, including 85 protein-coding genes, 37 tRNA genes, and eight rRNA genes. In the maximum-likelihood (ML) phylogenetic tree, *Pterygocalyx* clustered with the genus *Swertia*. This sequenced chloroplast genome of *P. volubilis* supports that *Pterygocalyx* belongs to subtribe Swertiinae.

*Pterygocalyx volubilis* Maxim., an annual herbaceous with twining stems, is the unique species of the genus *Pterygocalyx* (Gentianaceae), which is mainly distributed in temperate regions of East Asia (Ho and James 1995). *Pterygocalyx volubilis* is a traditional Chinese medicine, and its whole grass is used in the treatment of pulmonary tuberculosis and other conditions (Li et al. 2011). However, botanists hold different views on the belonging of this species (Chen et al. 1998). The chloroplast (cp) genome is very useful in plant systematics research due to its haploid nature, maternal inheritance, and highly conserved structures (Fu et al. 2016). Here, the complete cp genome of *P. volubilis* (Genbank accession number: MK993635) was sequenced on the Illumina HiSeq Platform, which will help to understand phylogeny of Gentianaceae.

The fresh, young leaves of *P. volubilis* were collected from Yuanshuoshan Mountain, Qinghai Province China (101.70°E, 36.93°N) and dried immediately by silica gels. Total genomic DNA of *P. volubilis* was extracted from the dried leaves (about 0.1 g) with a modified CTAB method (Doyle and Doyle 1987). The voucher specimen was kept in Herbarium of the Northwest Institute of Plateau Biology (HNWP, Wang2018003), Northwest Institute of Plateau Biology, Chinese Academy of Sciences. Genome sequencing was performed using the Illumina HiSeq Platform (Illumina, San Diego, CA) at Genepioneer Biotechnologies Inc., Nanjing, China. Approximately 6.74 GB of clean data were yielded. The trimmed reads were mainly assembled using SPAdes (Bankevich et al. 2012). The assembled genome was annotated using CpGAVAS (Liu et al. 2012).

The complete cp genome of *P. volubilis* is 154,365 bp in length with a typical quadripartite structure, containing a pair of inverted repeated (IR) regions (25,928 bp) that are separated by a large single copy (LSC) region of 84,033 bp, and a small single copy (SSC) region of 18,476 bp. The GC content of the whole cp genome was 35.87%. A total of 130 functional genes were annotated, including 85 protein-coding genes, 37 tRNA genes, and eight rRNA genes. The protein-coding genes, tRNA genes, and rRNA genes account for 66.38%, 28.46%, and 6.15% of all annotated genes, respectively.

The maximum-likelihood phylogenetic tree (ML tree) was generated based on the complete cp genome of *P. volubilis* and other species of the family Gentianaceae (Figure 1). Alignment was conducted using MAFFT (Katoh and Standley 2013). The phylogenetic tree was built using RAxML (Stamatakis 2014) with bootstrap set to 1000. The results showed that *P. volubilis* was closely related to the genus *Swertia*. This result supports the increasingly widespread view that *P. volubilis* belongs to subtribe Swertiinae (Yuan and Küpfer 1995; Chen et al. 1998; Xi et al. 2014; Ho and Liu 2015).

**Figure 1.**
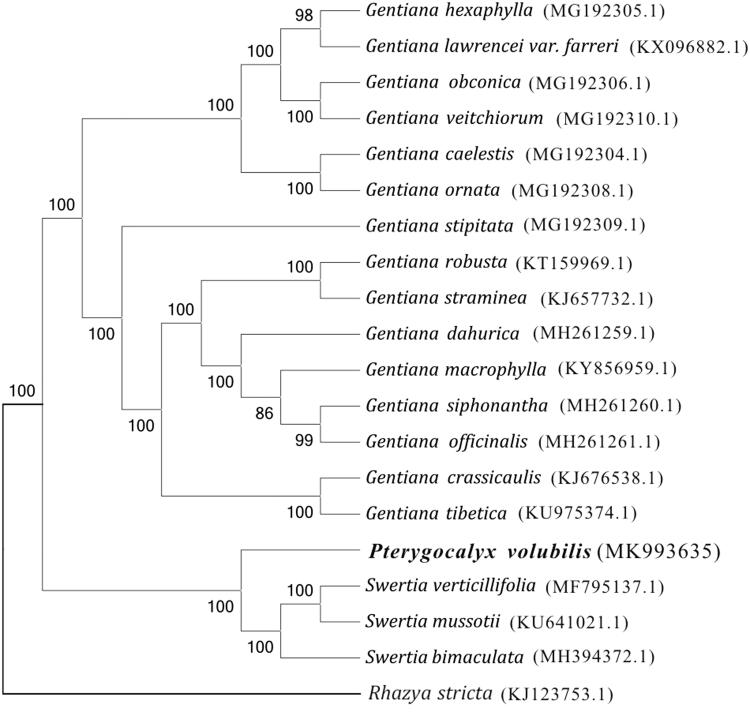
The ML tree based on 20 chloroplast genomes.
